# The Relationship Between Astronomical and Developmental Times Emerging in Modeling the Evolution of Agents

**DOI:** 10.3390/e26100887

**Published:** 2024-10-21

**Authors:** Alexander O. Gusev, Leonid M. Martyushev

**Affiliations:** 1 Technical Physics Department, Ural Federal University, Mira St. 19, 620062 Ekaterinburg, Russia; 2 Institute of Industrial Ecology, Russian Academy of Sciences, S Kovalevskoi St. 20a, 620219 Ekaterinburg, Russia

**Keywords:** Darwinian selection, entropy production, time, evolutionary computation, i8080 processor

## Abstract

The simplest evolutionary model for catching prey by an agent (predator) is considered. The simulation is performed on the basis of a software-emulated Intel i8080 processor. Maximizing the number of catches is chosen as the objective function. This function is associated with energy dissipation and developmental time. It is shown that during Darwinian evolution, agents with an initially a random set of processor commands subsequently acquire a successful catching skill. It is found that in the process of evolution, a logarithmic relationship between astronomical and developmental times arises in agents. This result is important for the ideas available in the literature about the close connection of such concepts as time, Darwinian selection, and the maximization of entropy production.

## 1. Introduction

In everyday life, as well as for most scientific, economic, and technological problems, we use astronomical time *t*. It is often also called chronological, extrinsic, physical, or clock time [1,2,3]. The reasons for the general use of this time *t* are largely historical, and, of course, also related to its simplicity and convenience. This time is uniform and absolute. Although these properties of time have been questioned by modern physics, for the overwhelming majority of “terrestrial tasks”, they are preserved with acceptable accuracy [1,2,3]. At the same time, sometimes astronomical time does not turn out to be a convenient quantity for the tasks existing in developmental and evolutionary biology (see, for example, [4,5,6,7,8,9,10,11,12,13]). For example, the duration of developmental periods, even in genetically similar species, can differ significantly in units of *t* due to the temperature of the environment or body, as well as its mass, etc. [6,7]. This leads to enormous difficulties when one wishes to discover and formulate any developmental law. One of the ways to overcome this problem is to reject extrinsic, absolute time and to use a time metric related to the processes inside the organism itself.

The search for such a metric has been going on for a long time [4,6,7,8,9,10,11,12]. Such a quantity is called developmental time *τ*. Other synonyms for this quantity are physiological, intrinsic, or biological time. Several developmental times have been proposed in the literature. Thus, in Ref. [4], the wound healing time was used as a unit of such time, and in Ref. [6], the time between the first and second cleavage divisions. Obviously, such metrics are not convenient and universal when considering processes occurring in different organisms. In this regard, developmental time metrics associated with metabolism and energy dissipation may be more useful [5,8,9,10,11,12]. In Ref. [12], a quantity related to mass-specific metabolic rate is used as such a metric. A generalization of this metric is carried out in Refs. [13,14,15,16], where it is proposed to use the density of entropy production by a body. As is known from the point of view of modern thermodynamics, entropy production is a fundamental quantity characterizing irreversible changes. This quantity can be experimentally determined and theoretically evaluated for processes related to life, development, and evolution. These reasons make the corresponding metric extremely important and convenient.

An important issue is finding the relationship between astronomical and developmental time. One of the first such relationships was proposed by Backman in 1943 on the basis of numerous studies of animal and plant growth [17]. According to it, the relationship between *τ* and *t* is logarithmic, i.e., *τ ∝* ln*t*. As a consequence, changes in the two times are recognized as d*τ ∝* d*t*/*t*. A modern, theoretical justification for such a relationship can be found in Ref. [13]. In this work, developmental time is determined through entropy production, and dimensional analysis is used, as well as the hypothesis of the universality of growth on different scales. As can be seen from the obtained dependence *τ*(*t*), the two times are related to each other nonlinearly. As a result, the use of its own intrinsic clock by the organism leads to a feeling of acceleration of astronomical time, and the older the organism is, the greater this acceleration becomes.

Previously, the relationship between developmental and astronomical times was considered only in the case of individual development and growth of an organism, i.e., researchers limited themselves to the processes of ontogenesis. In this case, either empirical regularities or any theoretical considerations related to ontogenesis were used. Another important process in the historical development of organisms is phylogenesis. This evolutionary process, occurring on much longer time scales, is subject to the well-known Darwinian triad: inheritance, variability, and selection. What relationship between developmental and astronomical times can arise from Darwinian evolution, and will it be functionally similar to the relationship found in ontogenesis? The purpose of this article is to consider these questions.

Evolutionary computation (modeling) is chosen as a method for solving the stated goal. The evolution of an agent based on Darwinian principles is considered; the objective function is the rate of capture of food randomly moving around.

## 2. Model

A model resembling the popular predator–prey model is used. There is an agent (predator) that must catch prey on a two-dimensional field of finite size. The predator is given a certain number of discrete movements, *T*, during which it can catch a different number of prey. All prey (food) are exactly the same. Only a single prey is presented on the field at a time, and only after it has been caught the next one appears in a random place in the field. The objective function of the agent’s evolution is to maximize the number of prey caught during *T*. Food does not evolve over time; its movement is jumpy and random, both in magnitude (a step of no more than *l*) and in direction. The agent’s movement is also jumpy. The distance (no more than *L*) and direction of the agent’s jump are set based on rules that emerge for the agent during the course of evolution itself. In other words, the agent’s movement rules are not programmed in advance; Darwinian evolution acts as the programmer.

For simplicity, the astronomical time *t* in the model is measured by the number of agent jumps (since the length of each jump can vary during the hunt, the speed of each jump can be different). As a result, if the very first agent exists on the field for time *t* from 0 to *T* (we will call this the first epoch), then its direct descendant exists from *T* to 2*T* (the second epoch), etc. We choose the number of prey caught by the agent during *T* as the developmental time *τ*. This choice can be justified by the fact that this quantity is proportional to the energy that the agent has received and eventually dissipated during the time *T*. As discussed in the Introduction, developmental time is currently usually associated with such dissipation. During the evolution of the agent, based on the objective function, dissipation, and *τ* are maximized. This is consistent with the well-known maximum entropy production principle, which is true for evolving arbitrary non-equilibrium systems (not necessarily living ones) [18,19,20,21]. In the present model, the uniform time is traditionally taken as time *t*, and the relation between this uniform time and time *τ* is to be established.

A set of commands (i8080 instructions) controlling the agent’s movement (a kind of genome) is exposed to evolutionary changes. Under the action of these commands, the current coordinates of the agent and the prey are read, and for the next time step, the movement vector of the agent is calculated and written. These coordinates are stored in special memory cells (the agent’s “sensors”). In this model, the sensor’s working principle is fixed and does not change in the course of evolution. The number of executable commands (executable genome length) of the agent is a parameter of the model, which we denote by Ψ. The contents of the genome (Ψ commands) are initially random. Agents are ranked by the number of catches made during time *T*. Outsider agents do not participate in further modeling; their genomes are destroyed. Successful agents continue to capture prey in the next epoch but do not produce offspring. Agents that are leaders in catching prey not only continue catching but also produce offspring; their genomes are passed on to offspring with a small fraction of changes (mutations) *δ* made. The more prey an agent catches during time *T*, the more offspring it leaves behind. Thus, the basic rules of Darwinian evolution are implemented: inheritance, variability, and selection. The chosen objective function ensures the selection of the fittest: more prey is caught, and, as a result, more resources (energy) are available to spend and leave more offspring behind.

Like any model, the present model is highly simplified and is not intended to reproduce any particular example of evolution observed in nature. If desired, the model can be easily expanded and complicated in various directions. In particular, taking into account the energy costs of the agent for executing control commands, moving across the field, and the time spent on the field without movement.

The main ideas of the constructed model are presented here. The technical details of the model implementation, as well as the simulation parameters used, are given in Appendix A.

## 3. Results and Discussion

Let us list the main, most interesting results.

**Figure 1:**
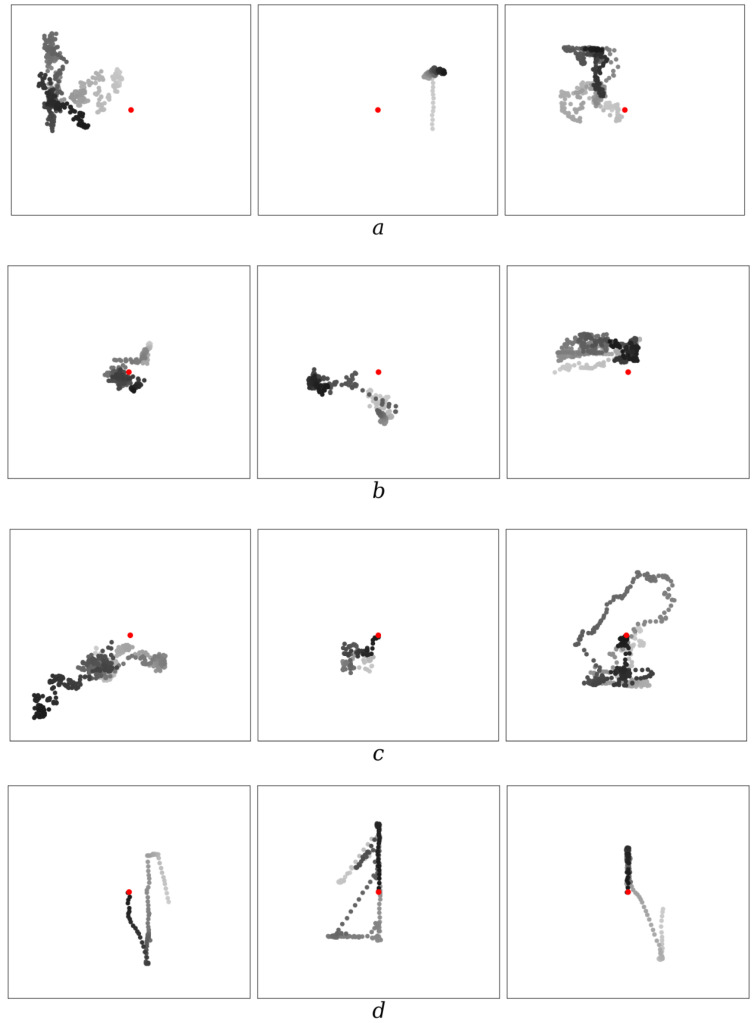
Examples of trajectories of the agent (predator) relative to the prey for different epochs of evolution: (**a**) 100 epoch, (**b**) 200 epoch, (**c**) 400 epoch, and (**d**) 800 epoch. The position of the prey, indicated by the red dot, is in the center (the reference frame is placed in it). The positions of the agent are shown by dots of varying degrees of blackness: from gray (corresponding to the beginning of the movement) to black (corresponding to the end of the movement). *δ* = 0.05 and Ψ =100.

1. Agents acquire the skill of catching in the process of evolution. A number of examples are presented in Figure 1. According to the presented results of modeling (see Figure 1), one hundred epochs of evolution are most often insufficient for acquiring the skill of catching. By the two-hundredth epoch, some agents begin to catch prey. The percentage of catches by agents increases by the four-hundredth epoch, and finally, with a number of epochs of eight hundred or more, agents begin to catch prey steadily and relatively quickly.

It is important to note that an agent who, in the process of evolution, has learned to catch prey moving along a random trajectory can also effectively catch prey that starts moving not randomly but, for example, along circular trajectories. Also, in the present evolutionary modeling, studies have been carried out on the possibility of the evolutionary acquisition of skills to effectively catch prey moving in a circular trajectory in a certain direction. As a result, after several hundred epochs of the Darwinian selection rules embedded in the model, it was possible to obtain predators that catch prey moving, for example, clockwise, and completely ignore prey moving counterclockwise. This result is quite unexpected and surprising since, in the model under consideration, the agent’s “sensors” always contain only the current coordinates of the agent and the prey. Therefore, the agent at each moment knows nothing about the system’s past. However, despite this, evolution “manages to teach” the agent to distinguish between different directions of the prey’s movement. As a result, in the program code that controls the catching of the agent, thanks to Darwinian principles, some representation of the past and the future is self-generated, i.e., there are signs of the most important property attributed to the concept of time.

**Figure 2:**
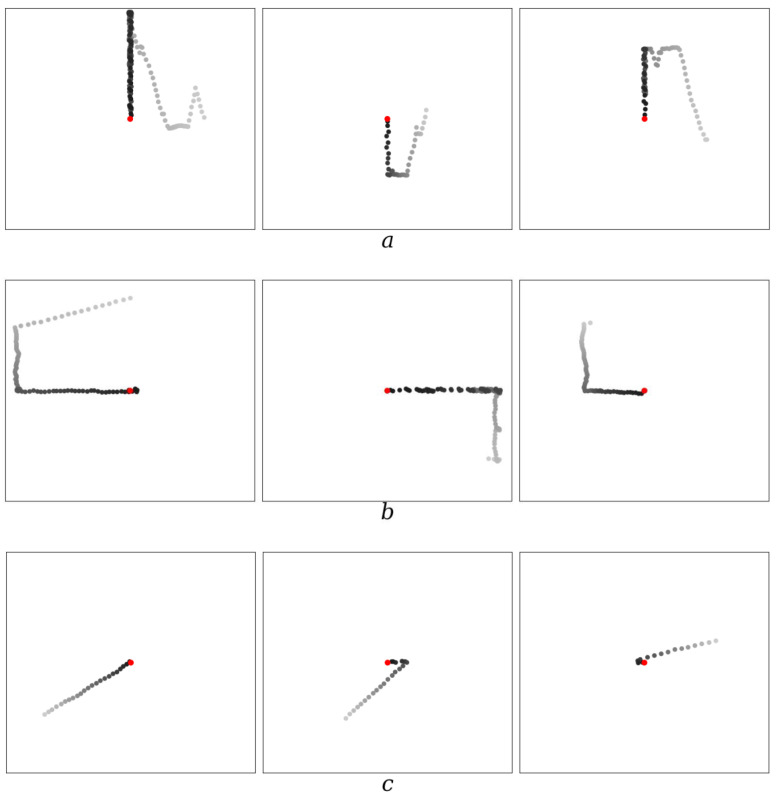
Examples of strategies of an agent (predator) catching prey on a 256 × 256 field. (**a**) Catching with the agent aligned vertically with the prey; (**b**) catching with the agent aligned horizontally with the prey; (**c**) catching prey by the agent along the shortest straight-line trajectory. The position of the prey, indicated by the red dot, is in the center (the reference frame is placed in it). The positions of the agent are shown by dots of varying degrees of blackness: from gray (corresponding to the beginning of the movement) to black (corresponding to the end of the movement). *δ* = 0.05 and Ψ = 100.

**Figure 3:**
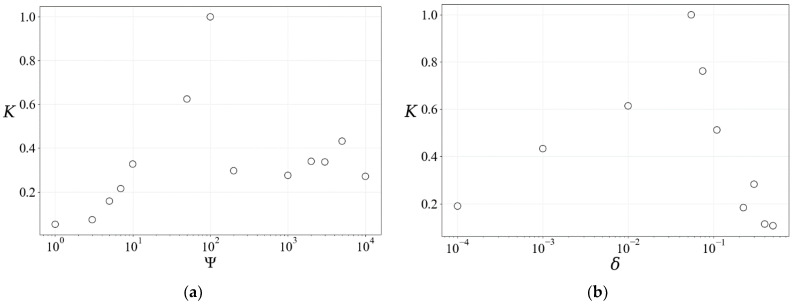
The success rate of prey capture by an agent (*K*) as a function of genome length, Ψ (**a**), and relative number of mutations, δ (**b**). *K* is taken as the ratio of the total number of captures per 10,000 epochs by an ensemble of 100 agents to the maximum value achieved during modeling. This coefficient gives an average and robust estimate of the success of the evolution of the catching skill of agents. In the calculations, we assumed *δ* = 0.05 for (**a**) and Ψ = 100 for (**b**).

**Figure 4:**
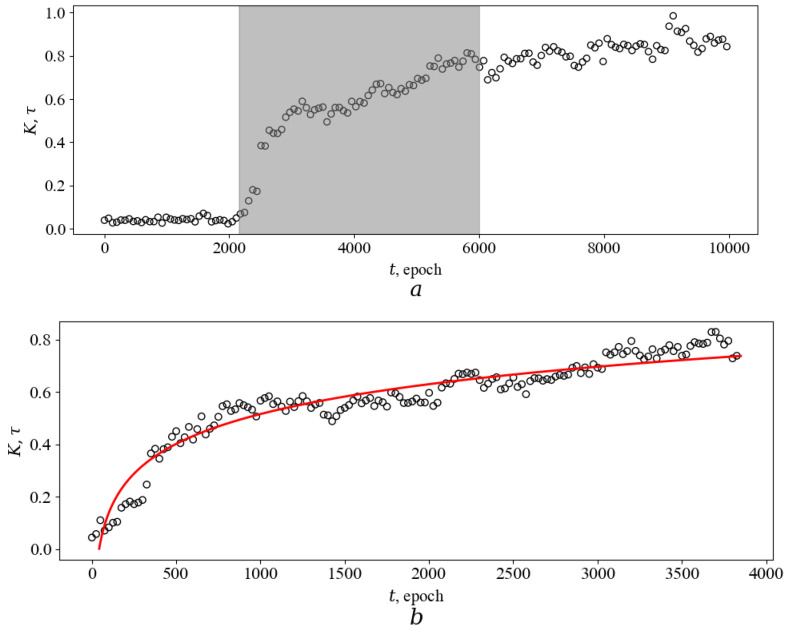
Evolution of an agent with one jump change. The success rate of prey capture by an agent (*K*) or development time *τ* as a function of astronomical time *t*. *K* is taken as the ratio of the total number of captures per one epoch by an ensemble of 100 agents to the maximum value achieved during evolution (10,000 epoch). *δ* = 0.05 and Ψ = 100. (**a**) Modeling result (the nonlinear part of the evolution is highlighted in gray. (**b**) The nonlinear part of the obtained dependence *τ*(*t*) (dots) and its fitting (red line) using the logarithmic function are presented in large detail. The logarithmic fitting is 0.16 × ln(*t*) − 0.62; the coefficient of determination *R*^2^ is 0.92.

**Figure 5:**
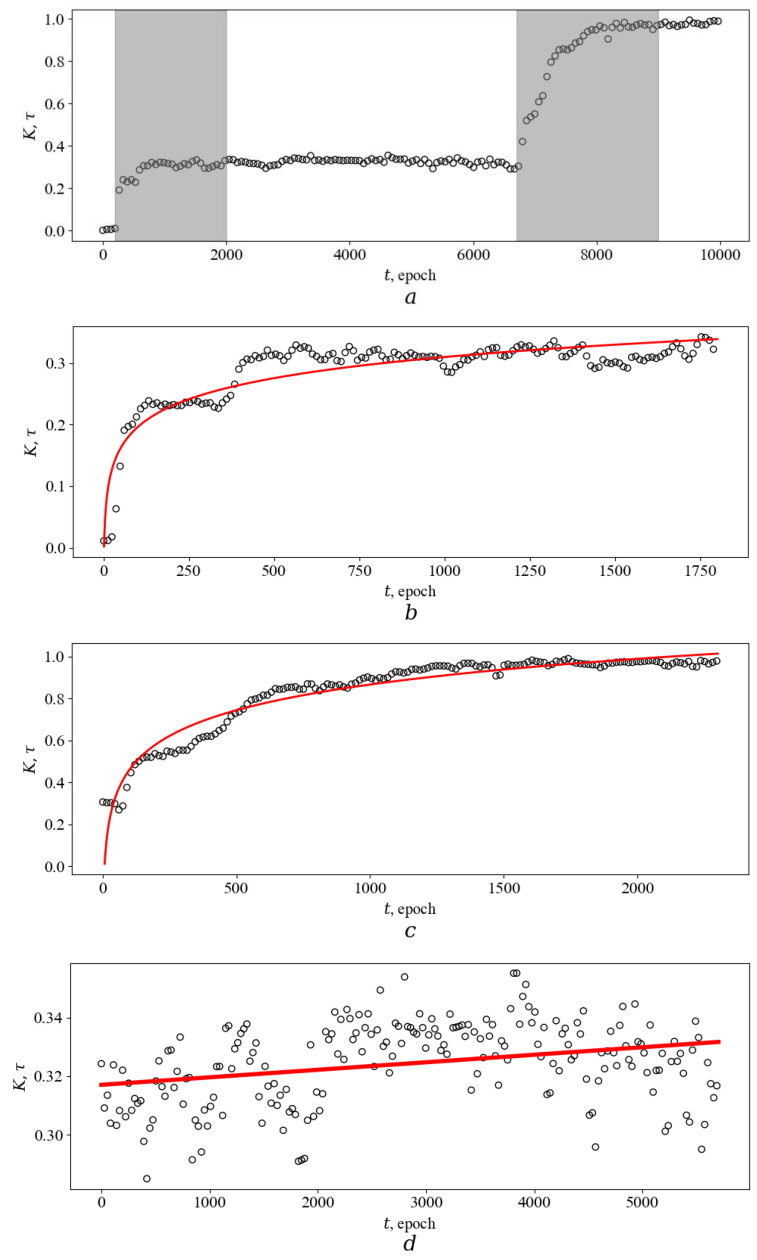
Evolution of an agent with two jump changes. The success rate of prey capture by an agent (*K*) or development time *τ* as a function of astronomical time *t*. *K* is taken as the ratio of the total number of captures per one epoch by an ensemble of 100 agents to the maximum value achieved during evolution (10,000 epoch). *δ* = 0.05 and Ψ = 100. (**a**) Modeling result (two nonlinear parts of the evolution are highlighted in gray. (**b**) The first nonlinear part of the obtained dependence *τ*(*t*) (dots) and its fitting (red line) using the logarithmic function are presented in large detail. The logarithmic fitting is 0.05 × ln(*t*) − 0.03, coefficient of determination, *R*^2^ is 0.80. (**c**) The second nonlinear part of the obtained dependence *τ*(*t*) (dots) and its fitting (red line) using the logarithmic function are presented in large detail. The logarithmic fitting is 0.18 × ln(*t*) − 0.36, coefficient of determination, R^2^ is 0.92. (**d**) The linear part of the obtained dependence *τ*(*t*) (dots) and its fitting (red line) using the linear model are presented in large detail. The linear fitting is 2.6 × 10^−6^ × *t* + 0.32. The slope, according to the calculation, is reliably not equal to zero: (2.6 ± 0.2) × 10^−6^.

2. In the process of evolution, agents can develop different catching strategies. A predator can align its coordinate with the coordinate of the prey vertically Figure 2a or horizontally Figure 2b, and then rapidly catch up with the prey in a straight line. Another, more optimal way of catching Figure 2c is to move towards the prey immediately along the shortest distance, in a straight line, regardless of the mutual position of predator and prey. The choice of strategy arises randomly in the course of evolution. Having arisen through mutation in an agent, this method of catching then begins to improve in the course of evolution from epoch to epoch.

3. The appearance of a predator’s catching skill, as well as the degree of mastery of this skill, depends significantly on the genome length Ψ (Figure 3a) and the number of mutations *δ* (Figure 3b). As can be seen from the figures, too small or too large values of Ψ and *δ* have a negative impact on the development of the catching skill. In short chains of the executable genome, evolutionarily useful changes, if they accidentally arise due to mutations, are rather quickly suppressed (distorted) by subsequent random mutations. In long chains, apparently, the changes introduced by mutations are not enough to cause significant changes, leading to a significant improvement in the agent’s catching skills. The relative number of random changes per unit of genome length should have some optimal, average value for evolution to proceed most effectively. Note also that, according to calculations, the improvement of a predator’s catching skill is significantly more effective with moving prey than with resting prey. Therefore, changes not only in the genome but also in the agent’s “sensors” (registers) are important for the appearance of a useful feature. This property of the model echoes an observation from sports pedagogy: teaching young football players the technique of kicking a moving ball with the non-striking (non-supporting) foot occurs more quickly and effectively compared to kicking a stationary ball [22].

4. Figure 4 and Figure 5 show the characteristic behavior of the relative number of prey caught by a predator during one epoch *T* as a function of epoch number. As discussed above, this graph essentially represents the dependence of developmental time *τ* on astronomical time *t*. As can be seen, the presented dependencies have two characteristic parts. In the first part, there is a very weak linear dependence of *τ* on *t* (see, e.g., Figure 5d). Here, predators are not undergoing any important evolutionary changes and are not improving their catching skills. In the second part, there is a nonlinear, often jump-like increase in *τ* depending on *t*. This behavior is a consequence of the occurrence of some favorable mutations in the agent’s genome, which led to the appearance of useful skills for successfully catching prey. The rapid increases in hunting efficiency resemble phase transitions, especially kinetic (nonequilibrium) transitions such as breakdown in a dielectric or destruction of a body if the number of cracks in it exceeds a certain threshold value. Apparently, such an analogy can be useful in describing and explaining the observed. Indeed, the continuous accumulation of mutations in the genome, reaching a certain critical value, must inevitably lead to a bifurcation in the agent’s behavior. The result of this bifurcation is either a significant deterioration of hunting efficiency or a significant jump-like improvement. Due to selection, agents of the first type die, and those of the second type increase their numbers in an avalanche-like manner. Owing to the large number of simultaneously evolving agents (large statistics), unsuccessful mutations do not have a visible effect on the average ensemble characteristics of type *K*. On the contrary, successful mutations, according to the principle of positive feedback, spread to many agents and become statistically significant, and, as a result, a jump-like increase in *K* is observed in Figure 4 and Figure 5, respectively.

**Figure 6:**
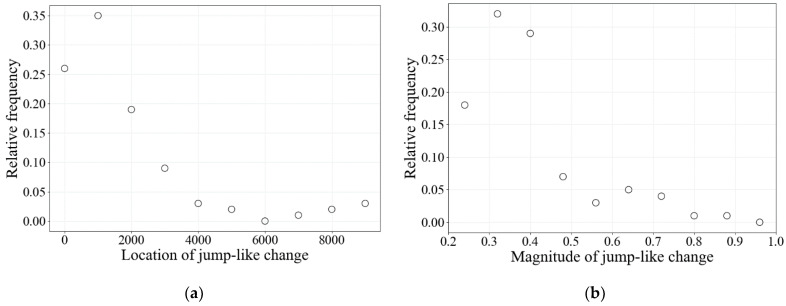
The relative frequency of occurrence of some values of (**a**) location and (**b**) jump magnitude at jump-like changes *K*. *δ* = 0.05 and Ψ = 100.

According to the calculations, the magnitude and location of the occurrence of these jump-like changes are quite random. As shown in Figure 6a,b, jump-like changes are most likely to occur during the first 2000 epochs, and the most probable magnitudes of jump-like changes are in the range from 0.2 to 0.4. Note that these values may change if the model parameters *δ* and Ψ are changed. As a result of modeling, it was possible to observe from one to four jumps during the evolutionary time considered in the paper.

The nonlinear dependence *τ*(*t*), as can be seen from the results presented in Figure 4 and Figure 5, is described quite well by the logarithmic dependence. Indeed, the goodness of fit of the logarithmic model has a correlation coefficient of at least 0.8. Using statistical tests, it is difficult to make a choice between different mathematical models suitable for describing *τ*(*t*). Indeed, consider the dependence in Figure 4b (*R*^2^ = 0.92). For this dependence, the best power approximation (0.0001 × *t*)^0.36^ has *R*^2^ equal to 0.90, and the best hyperbolic model 0.86 × *t*/(*t* + 666.5) has *R*^2^ equal to 0.91. The behavior of the squared residuals between the obtained data and the model predictions over time also does not allow us to choose in favor of any model since one model has slightly smaller values on some time intervals and the other on another. Obviously, it is always possible to invent more complex (e.g., exponential-based) models, as well as models containing a larger number of fitting parameters. These models are likely to be able to better statistically describe the observed behavior of *τ*(*t*). Therefore, when selecting a model, one should, first of all, be based on information related to the essence of the phenomenon that is supposed to be described (of course, such a choice should be made on a set of models that do not contradict the available data and have proved themselves well enough by the results of statistical tests).

Let us perform such an analysis. (1) According to the calculations obtained in our work, the dependence of *τ*(*t*) usually does not reach a plateau (there is an increase without bound). For example, for the practically horizontal part shown in Figure 5a, the linear equation has the form: 2.6 × 10^−6^ × *t* + 0.32, where the slope, according to the calculation, is reliably not equal to zero (2.6 ± 0.2) × 10^−6^ (см. Figure 5d). (Also, the results of evolutionary experiments on bacteria [23,24,25] indicate the absence of asymptotic growth, which will be discussed in detail below). As a consequence, it is necessary to exclude models with asymptotic growth, such as hyperbolic ones, from consideration. (2) The parameter *K* characterizes the number of prey caught by the agent per unit of time. Obviously, at the zero moment of time, this parameter is meaningless, so this must be taken into account when choosing a mathematical model. Power approximation, as well as exponential models demonstrating S-shaped behavior (e.g., logistic, Weibull, Gompertz, etc.), have some definite value at *t* = 0. On the contrary, the logarithmic function is undefined at *t* = 0 and this is its advantage. (3) The logarithmic function is used in the work to describe single jump-like changes (see Figure 5b,c). As mentioned earlier, there can be several jumps during evolution. We were able to observe up to four jumps during a computer simulation of evolution during 10,000 epochs. If the agent’s genome is sufficiently complex (in particular, Ψ is large), then the following is most likely to be assumed: if the evolution time is several orders of magnitude greater than 10,000, the number of jumps will be much larger, but the type of the curve as a whole will not change and will resemble that shown in Figure 4 and Figure 5. Obviously, in this case, the behavior of *K* over the entire interval *t* from zero to infinity can also be approximated by a logarithmic function. The logarithmic function can describe the dependence of *τ* on *t* both for a single jump at a relatively small time interval *t* and for a set of successive jumps when considering large time intervals.

The above arguments allow us to conclude that the obtained dependencies *τ*(*t*) are preferable to be described by a logarithmic model.

The emerging nonlinearity is a non-obvious and interesting evolutionary property of the agent. Indeed, the agent hunts a prey that changes its coordinates uniformly in time *t*. Mutations in the agent’s genome also occur uniformly. Thus, the agent is in an environment with continuous and uniform changes in time. However, due to Darwinian selection, it develops discontinuous and non-uniform properties.

It is important to note here that the dependences presented in Figure 4 and Figure 5 are qualitatively similar to the dependences of fitness (corresponding in meaning to *K*) on astronomical time, which were discovered in the well-known long-term evolutionary experiment on *Escherichia coli* bacteria [23,24,25]. As was obtained in these experiments, the increase in fitness (which the authors estimated by the rate of bacterial reproduction in comparison with the original, ancestral strain) sharply increases over time and then slows down but does not reach a plateau. Experimental bacteria constantly accumulate useful mutations, steadily increasing their adaptability in an environment in which the same conditions are maintained all the time. As a consequence, as the authors note [24], the real growth of adaptability cannot be described by models that demonstrate asymptotic growth to some constant value, but it is necessary to use models that demonstrate a weak permanent increase. The logarithmic model used in the work is just such a model.

## 4. Conclusions

A computer model demonstrating the evolutionary development of a predator’s ability to catch prey has been developed and used. The principles of the model are based on the famous Darwin triad, which has once again demonstrated its amazing power. Indeed, without any a priori knowledge of where the information from the senses will be sent and without any rules for processing this information, thanks to natural selection, the agent develops an effective algorithm for catching prey. This algorithm turns out to be extremely resistant to random changes introduced by mutations and universal since an agent trained on random movements of the prey turns out to be effective in catching prey that moves differently.

The principles of evolutionary modeling, which are the basis of this work, are a kind of “invisible programmer” (a blind watchmaker, in the terminology of R. Dawkins), creating a working product that gradually improves its characteristics from epoch to epoch. From the point of view of an external observer, the emerging code is absolutely incomprehensible since the “programmer” does not use a classical approach in which a logically thought-out simple algorithm is meticulously and error-free implemented. Each time, the Darwinian programmer initially takes some chaotic set of commands, and from them, point by point, changing separate commands adjusts the chaos to fulfill the goal (to catch the prey as quickly as possible, in our case). In our case, this programmer works in an extremely low-level language, the language of machine codes of the processor. This, despite the random, “atomic” nature of changes in the genome, leads to executable code sequences and, ultimately, to the emergence of an effective catching algorithm. The system we have considered is representative of machine learning (ML) systems. However, unlike most ML systems, we use discrete adjustable internal parameters (discrete set of i8080 processor commands), not continuous ones (float numbers), and, as a result, we do not use gradient descent in parameter optimization. In addition to this, the mutation mechanism is also a discrete point. Despite these differences, our system begins to catch effectively under the influence of evolution. It is also noteworthy that the genome length and the relative number of mutations (the hyperparameters of our ML system) significantly affect the rate of evolution (convergence of the optimization algorithm), and some of their optimal values are detected. With these values, the agent learns to catch significantly more effectively compared to its other values.

An important result of the modeling is the obtained logarithmic relationship between astronomical and developmental times. The first time is reversible and uniform. This time originally arose in mechanics, and it is inseparably connected to motion. From the point of view of modern physics, such time can be excluded from the number of the most important fundamental variables [3]. The second time, irreversible and non-uniform, arose in thermodynamics and biology. Unlike astronomical time, developmental time is the most important characteristic of the system, and it can be identified with the very existence of the system both in its individual development from birth to death and in its historical development from ancestors to descendants. While for development during ontogenesis, the logarithmic relationship between the two times has been written about many times, for development during phylogenesis, the similar relationship obtained in the present evolutionary modeling is a new and important result that requires further theoretical and experimental verification. This result is a manifestation of the close connection between Darwinian evolution and the maximization of entropy production [20,21,26,27], as well as between entropy production and time [13,14,15,16].

The problem under consideration, due to the generality of its formulation and the method chosen, is of interest not only to evolutionary biology. Indeed, adherence to Darwinian principles can be observed in the development of technology, society, economics, etc. (see, for example, [28]). For example, as a possible application of the modeling results, one can consider the development of a commercial firm maximizing profit.

## Figures and Tables

**Figure A1:**
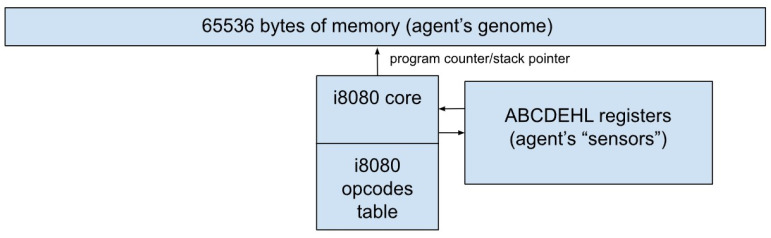
Block diagram of the i8080 processor used in this model as the agent’s “brain”.

## Data Availability

Data are contained within the article.

## Appendix A

Here, we present the peculiarities of the computer realization of the model. The use of this modeling approach was initiated by Refs. [29,30].

The agent’s instruction set is based on a software-emulated Intel 8080 processor. A slightly modified version of this emulator is used (https://github.com/nav97/Intel-8080-Emulator, accessed on 10 January 2022). The instruction table (opcodes) of the i8080 processor is well known (see, for example, https://pastraiser.com/cpu/i8080/i8080_opcodes.html, accessed on 10 January 2022). The block diagram of the processor is shown in Figure A1. We will use the instruction table with a slight modification: reset, data input/output, and interrupt control opcodes (RST*, IN, OUT, EI, DI) will be ignored and executed as NOP (no operation). Such modifications are made in order to use only the logical and arithmetic powers of the processor, as well as memory instructions. The maximum possible agent genome is 65,536 bytes of memory provided to the i8080 processor for storing instructions. The initial state before calculating the movement coordinate is determined as follows: the program pointer PC is set as PC = 0 × 8000 and further increases and the stack pointer is set as SP = 0 × 7FFF and further decreases. The coordinates of the prey and the agent are saved in the processor registers. Registers B and C are used to store the *x* and *y* coordinates of the prey, and registers D and E are used to store the *x* and *y* coordinates of the agent. The remaining registers are reset to zero before starting execution. Next, Ψ instructions (opcodes) are executed, after which the execution stops, and data is unloaded from register A, which is interpreted as the vector of movement of the agent on the field.

The reason for choosing the Intel i8080 processor as the basis for modeling is the availability of its computer-implemented simulation in the public domain and its relative simplicity. The most important reason for using such a processor is that its instruction set is, by definition, Turing-complete, and thus, with the help of these instructions, one can theoretically realize any calculation and algorithm in a finite amount of time. The choice of registers as agent’s sensors is explained by the fact that during any execution of processor commands, registers will always be involved.

The algorithm for making the next jump by the agent is as follows:

1. Registers B, C, D, and E are loaded with information about the coordinates of the agent and the prey. Registers B and C contain the *x* and *y* coordinates of the prey’s position on the Cartesian field, and registers D and E contain the *x* and *y* coordinates of the agent on this field at the moment.
2. The remaining registers and processor flags are reset to zero, and the counters are set to the initial state.
3. The processor starts the execution of Ψ memory instructions. Once Ψ instructions have been executed, further instruction execution by the processor is stopped.
4. Data is read from register A, which is interpreted as the coordinates of the new position of the agent. This happens as follows. Register A is an 8-bit register; these 8 bits are divided into a pair of 4-bit “words”. The first bit of each 4-bit word is treated as a sign bit (0 is positive, 1 is negative). The remaining 3 bits of the 4-bit word are used as significant bits. This produces two numbers in the range of −8 to +8, which are the *x* and *y* coordinates of the jump vector. Thus, the agent can jump a certain number of cells in an arbitrary direction. In this case, the jump length *L* is no more than 11 (the length of the diagonal vector, each component of which is 8 by absolute value).
5. The algorithm is repeated until *T* jumps are reached.

Unlike the agent, its prey makes jumps that are random in direction and magnitude, with a step size *l* of no more than one cell in an 8-connected neighborhood. The modeling in this paper takes place on a square field of 256 by 256 cells. Making jumps as described above, the agent and the prey move across the field. The prey is considered caught if the distance between the prey and the agent is no more than three. In total, the agent is given *T* jumps across the field to hunt (in this paper, *T* = 1000 was assumed). During this time, it can catch the prey as many times as it can. At the very beginning of the simulation and after each catch, the positions of the agent and the prey on the field are set randomly.

Catching as many prey as possible in *T* jumps is the objective function of evolution. The more prey is caught, the more valuable this agent is for evolution and the more descendants it will leave. Simulation of evolution with selection of the best agent is carried out simultaneously on an ensemble (or batch if we use ML terminology) of 100 parallel and independently executed algorithms. This is done in order to gain an advantage in multithreaded computation on a multi-core processor and statistically “smooth” the obtained results. The genomes of the agents have a length of 65,536 but are always executed for the length Ψ. The content of the genomes at the initial moment of time is randomly generated, but the same for all 100 agents. One hundred agents of the ensemble after time *T* are ranked by the number of captures from the smallest to the largest and those *n* agents whose number of captures exceeds 0.75 quantiles of captures of agents of the ensemble are selected. These *n* agents are considered “successful” and their genome is passed on to *n* agents who will hunt in the next round. The remaining 100 − *n* participants in the next round are the descendants of the three leaders in the number of captures. Moreover, 0.5 × (100 − *n*) descendants are selected from the agent who has made the most captures, 0.3 × (100 − *n*) from the agent in second place, and 0.2 × (100 − *n*) from the agent in third place. All descendants are generated from the parent genome slightly modified (mutated). This is done by changing the fraction of bytes *δ* of the whole genome to other randomly generated bytes. In cases when *n* is equal from 0 to 3, there is no procedure for selecting the best agents and generating descendants: all 100 agents unconditionally pass to the next round of the predator-prey game without changing their genomes.

The built-in random number generator from the Numpy (version number 2.1.1) package (Python programming language, version number 3.10.11) was used in this work. The cycle, including catching, selection of agents most successful in catching, and appearance of new descendants-agents from them, is repeated 10,000 times (i.e., the number of epochs is 10,000). After this, the evolutionary game ends.
